# Mitigating saturation effects in rice nitrogen estimation using Dualex measurements and machine learning

**DOI:** 10.3389/fpls.2024.1518272

**Published:** 2024-12-16

**Authors:** Peihua Shi, Yuan Wang, Congfei Yin, Kaiqing Fan, Yinfei Qian, Gui Chen

**Affiliations:** ^1^ Department of Agronomy and Horticulture, Jiangsu Vocational College of Agriculture and Forestry, Jurong, China; ^2^ State Key Laboratory of Soil and Sustainable Agriculture, Changshu National Agro-Ecosystem Observation and Research Station, Institute of Soil Science, Chinese Academy of Sciences, Nanjing, China; ^3^ Soil and Fertilizer & Resources and Environmental Institute, Jiangxi Academy of Agricultural Sciences, Nanchang, China; ^4^ Institute of Biotechnology, Jiaxing Academy of Agricultural Science, Jiaxing, China

**Keywords:** rice nitrogen estimation, Dualex measurements, saturation effect, incremental analysis, machine learning, nitrogen balance index, SHAP analysis

## Abstract

Nitrogen is essential for rice growth and yield formation, but traditional methods for assessing nitrogen status are often labor-intensive and unreliable at high nitrogen levels due to saturation effects. This study evaluates the effectiveness of flavonoid content (Flav) and the Nitrogen Balance Index (NBI), measured using a Dualex sensor and combined with machine learning models, for precise nitrogen status estimation in rice. Field experiments involving 15 rice varieties under varying nitrogen application levels collected Dualex measurements of chlorophyll (Chl), Flav, and NBI from the top five leaves at key growth stages. Incremental analysis was performed to quantify saturation effects, revealing that chlorophyll measurements saturated at high nitrogen levels, limiting their reliability. In contrast, Flav and NBI remained sensitive across all nitrogen levels, accurately reflecting nitrogen status. Machine learning models, particularly random forest and extreme gradient boosting, achieved high prediction accuracy for leaf and plant nitrogen concentrations (R^2^ > 0.82), with SHAP analysis identifying NBI and Flav from the top two leaves as the most influential predictors. By combining Flav and NBI measurements with machine learning, this approach effectively overcomes chlorophyll-based saturation limitations, enabling precise nitrogen estimation across diverse conditions and offering practical solutions for improved nitrogen management in rice cultivation.

## 1 Introduction

Nitrogen is a fundamental nutrient for rice growth and yield formation, playing a crucial role in processes such as photosynthesis, protein synthesis, and enzyme activity (Liu et al., 2021; Luo et al., 2021). Effective nitrogen management not only boosts rice yield and quality but also reduces environmental pollution from excessive fertilizer use, thereby supporting sustainable agriculture (Guo et al., 2020; Cai et al., 2023). Therefore, accurately monitoring the nitrogen nutritional status of rice is vital for developing effective fertilization strategies.

Traditional nitrogen assessment methods largely depend on laboratory analyses, which are often cumbersome, time-consuming, and expensive, making them impractical for large-scale, real-time monitoring (Berger et al., 2020). To address the need for fast, non-destructive measurements, devices such as the SPAD chlorophyll meter have been widely used (Tan et al., 2021; Singh et al., 2022). These tools estimate chlorophyll content by analyzing the optical properties of leaves, thus providing an indirect measure of nitrogen levels. However, chlorophyll measurements often show saturation effects at high nitrogen levels, limiting their ability to accurately capture differences in nitrogen content (Jiang et al., 2021; Gao et al., 2024). This drawback affects their precision under high-nitrogen conditions, hindering accurate nitrogen management.

To overcome these limitations, multispectral and hyperspectral remote sensing technologies at the canopy level have been introduced for agricultural nitrogen monitoring (Fu et al., 2021; Qian et al., 2022). These methods utilize the reflectance characteristics of vegetation across various spectral bands to develop indices such as the Normalized Difference Vegetation Index (NDVI) and the Optimized Soil-Adjusted Vegetation Index (OSAVI). These indices help estimate crop nitrogen content and biomass (Qian et al., 2022; Yang et al., 2023; Li et al., 2024). Although remote sensing is beneficial for large-scale, non-contact monitoring, it is also subject to limitations. Data reliability can be affected by weather conditions, observation angles, and variations in light, leading to inconsistencies (Ma et al., 2020; Wu et al., 2023). Furthermore, at high biomass or nitrogen levels, these indices may also exhibit saturation effects, resulting in reduced accuracy in nitrogen estimation (Zhang et al., 2019b; Chang et al., 2022).

Saturation effects present a major challenge in nitrogen estimation. When measurements saturate at high nitrogen levels, distinguishing between different nitrogen statuses becomes difficult, regardless of whether chlorophyll content or canopy-level indices are used. This leads to reduced accuracy in high-nitrogen conditions, complicating the implementation of precise nitrogen management. Therefore, new approaches that can mitigate or eliminate saturation effects are urgently needed to enhance the accuracy and reliability of nitrogen estimation.

Flavonoids (Flav) are a diverse group of phenolic compounds that are abundantly present in plants and play key roles in their responses to biotic and abiotic stresses (Gitelson et al., 2017; Hamdane et al., 2023). Under stress conditions, such as nitrogen deficiency, Flav levels increase, making them reliable indicators of nitrogen stress (Li et al., 2021; Waqas et al., 2023). The portable Dualex sensor facilitates the simultaneous measurement of chlorophyll (Chl) and Flav in leaves, allowing the calculation of the Nitrogen Balance Index (NBI), which is derived as the ratio of Chl to Flav (Cerovic et al., 2012, 2015). Measurements of Chl and Flav have been effective in differentiating genotypic responses to nitrogen and water stress in wheat (Raya-Sereno et al., 2024). Studies have shown that the Dualex sensor maintains a stronger linear correlation with actual chlorophyll concentration than devices like SPAD-502 and CCM-200, especially at higher chlorophyll levels. It also demonstrates higher reliability in tests on crops such as maize, soybean, and wheat, being less susceptible to the effects of uneven chlorophyll distribution (Dong et al., 2019, 2022). Dong et al. (2021) successfully estimated the Nitrogen Nutrition Index (NNI) in maize across various growth stages by applying multiple linear regression models that integrated Dualex readings with environmental variables. Overall, Dualex measurements prove to be valuable in estimating plant chlorophyll and nitrogen content, as well as in distinguishing different levels of nitrogen and water stress, particularly maintaining high sensitivity even under high nitrogen conditions (Rubo and Zinkernagel, 2022). However, there is still a lack of comprehensive research on the role of Flav and NBI in rice nitrogen estimation and their performance under varying nitrogen conditions.

Nitrogen distribution within rice plants shows variability across different leaf positions (Chang et al., 2019; Ouyang et al., 2021). Karaca et al. (2023) highlighted that optical sensor data collected from various leaf positions can improve the estimation of crop nitrogen status. In crops like melon and sweet pepper, data from lower leaves demonstrated superior predictive performance at certain growth stages. Another study reported that Dualex sensor measurements across different leaf positions performed similarly when estimating the NNI. However, data from the third fully expanded Leaf From the Top (LFT) often showed higher predictive accuracy, especially during the later growth stages of maize (Dong et al., 2021). Some studies also suggest that variations in leaf layers or specific measurement positions do not significantly impact the assessment of nitrogen content in wheat when using spectral reflectance (Röll et al., 2019). In rice, SPAD readings taken from the upper-middle section of the fourth LFT have been found to provide more accurate and stable reflections of nitrogen status (Yuan et al., 2016). These findings suggest that relying on a single leaf position may not comprehensively capture the overall nitrogen status of the plant. Therefore, using multi-leaf, multi-indicator data could improve nitrogen diagnosis accuracy. However, integrating data from multiple leaf positions and indicators to build accurate nitrogen estimation models remains a challenge that requires further investigation.

Machine learning models are particularly effective at handling complex nonlinear relationships and multivariate data, making them well-suited for agricultural data analysis and prediction tasks (Shi et al., 2021; Huang et al., 2023; Mandal et al., 2024). Combining machine learning with Dualex measurements offers the potential to enhance nitrogen estimation accuracy and mitigate saturation effects. Based on this framework, our study systematically investigates the relationships between Dualex measurements and rice nitrogen indicators through multi-location, multi-variety, and multi-leaf-position testing. We assess the effectiveness of Flav and NBI in reducing saturation effects at high nitrogen levels. We also develop rice nitrogen nutritional indicator estimation models by integrating machine learning, focusing on optimizing feature selection and model structure to enhance interpretability and practical application. By addressing the limitations of traditional methods under high nitrogen conditions and extending applicability across different growth stages, we aim to ultimately improve the efficiency and sustainability of agricultural production.

## 2 Materials and methods

### 2.1 Study area and experimental design

Field experiments were conducted across Jiangsu, Zhejiang, and Jiangxi provinces in China. These experiments followed a randomized block design, incorporating 3 to 6 different nitrogen application rates, each with 3 to 4 replicates. A total of 15 rice varieties were tested, including 10 japonica, 4 indica, and 1 indica-japonica hybrid, covering a range of regions and cultivation systems. Basic details of these varieties are shown in **Table 1**. Except for variations in nitrogen application, all other agronomic practices followed local conventional standards.

**Table 1:** Summary of field experiments and nitrogen treatments.

No.	Varieties	Subspecies	Experimental Site	Growth Period	Nitrogen Rate
1	NG3908	japonica	Nanjing	May–Oct	0, 60, 120, 180, 240, 300
2	NG5055	japonica	Nanjing	May–Oct	0, 100, 150, 200, 250, 300
3	NG46	japonica	Zhenjiang	May–Oct	0, 60, 120, 180, 240, 300
4	YG13	japonica	Zhenjiang	May–Oct	0, 75, 150, 225,300
5	NG3	japonica	Zhenjiang	May–Oct	0, 75, 150, 225,300
6	JH218	japonica	Jiaxing	May–Oct	0, 80, 120, 140, 160
7	JYZK6	japonica	Jiaxing	May–Oct	0, 200, 300
8	XS14	japonica	Jiaxing	May–Oct	0, 100, 200
9	YY15	Hybrid^1^	Jiaxing	May–Oct	0, 189, 230, 270
10	J67	japonica	Jiaxing	May–Oct	0, 127, 169
11	HHZ	indica	Yichun	Jun–Oct	0, 135, 180, 225
12	MXXZ	indica	Yichun	Jun–Oct	0, 135, 180, 225
13	CY5	japonica	Yichun	Jun–Oct	0, 135, 180, 225
14	TYXZ	indica	Yichun	Jun–Oct	0, 135, 180, 225
15	TYHZ	indica	Yichun	Jun–Oct	0, 135, 180, 225

^1^This rice variety is an indica-japonica hybrid bred by the Ningbo Academy of Agricultural Sciences.

### 2.2 Data collection

Samples were obtained every 10-15 days from the Tillering (TI) stage to the Heading (HD) stage, corresponding to key growth stages: TI, Stem Elongation (SE), Panicle Initiation (PI), and HD. Due to differences in growth durations among the rice varieties, some early-maturing varieties had entered the Grain Filling (GF) stage when most varieties were at HD. Consequently, a small number of samples were collected during the GF stage for these varieties. At each sampling time, 3 to 6 hills of uniformly growing plants were destructively sampled from each plot. Samples were separated into leaves, sheaths, and panicles, then oven-dried at 105°C for 30 minutes, followed by 75°C until a constant weight was achieved. Nitrogen concentration was determined using the Kjeldahl method.

The NNI was derived by calculating the ratio of Plant Nitrogen Concentration (PNC) to the Critical Nitrogen concentration (N_c_). NNI values greater than 1 indicate nitrogen surplus, 1 indicates sufficiency, and less than 1 indicates deficiency.


$$NNI=\frac{PNC}{N_{c}}$$

where N_c_ is calculated using the nitrogen dilution curve established from our dataset:


$$N_{c}=3.44\times W^{-0.43}$$

where W represents the above-ground dry matter (t·ha^-^¹). The parameters (3.44 and -0.43) were derived from our experimental data using the method described by Justes et al. (1994). This method involves plotting plant nitrogen concentration against biomass and fitting a power function to the minimum nitrogen concentration observed at each biomass level, representing the N_c_ required for optimal growth.

On the same day as the destructive sampling, 10 to 20 rice plants were selected from each plot to measure the 1-5 LFT using a Dualex Scientific+ instrument (Force-A Inc., France). Each leaf was measured at three positions: middle, upper one-third, and lower one-third, and the average value was calculated for that leaf. The Dualex Scientific+ is a portable optical sensor designed for rapid, non-destructive detection of various physiological parameters in plant leaves. By measuring the transmission and reflection of specific wavelengths of light, it provides readings for Chl, Flav, Anthocyanin content (Anth), and the NBI. NBI is the ratio of Chl to Flav, serves as an indirect measure of the plant’s nitrogen nutritional status. Although the Dualex Scientific+ instrument also measures Anth, preliminary analyses indicated that anthocyanin levels in rice leaves were consistently low and showed minimal variation across treatments and growth stages. Consequently, Anth was not included in the subsequent analyses.

During early growth stages, such as the TI stage, it was sometimes not possible to identify five fully expanded leaves in a shoot due to the limited number of leaves. In these cases, we measured at least the 1-4 LFT and calculated the average values. This average was then used as the value for the 5 LFT. This data preprocessing step ensured consistency across samples and prevented errors due to unequal data when applying machine learning models.

### 2.3 Modeling methods

The study employed several modeling approaches including Simple Linear Regression (LR), Quadratic Curve Regression (QCR), Partial Least Squares Regression (PLS), and four machine learning models—Support Vector Regression (SVR), Random Forest (RF), Extreme Gradient Boosting (XGB), and Neural Network (NN)—to estimate rice Leaf Nitrogen Concentration (LNC), PNC, and NNI. The hyperparameters for each machine learning model were selected based on preliminary analyses that involved testing various parameter combinations using grid search and cross-validation. This approach helped balance model complexity and performance, ensuring robust generalization. By comparing the performance of these models, their applicability in estimating rice nitrogen nutrition indices was evaluated.

#### 2.3.1 Simple linear regression and quadratic curve regression

Initial analyses used LR and QCR to examine the relationships between individual Dualex measurements (Chl, Flav, NBI) and nitrogen indices (LNC, PNC, NNI) to gain a preliminary understanding of variable interactions and fitting effectiveness.

#### 2.3.2 Partial least squares regression

PLS regression, a linear method suitable for multivariate, highly correlated data, was employed. It extracts latent variables that maximize the covariance between independent and dependent variables. In this study, the PLS model used two principal components to balance complexity and explanatory power.

#### 2.3.3 Support vector regression

SVR, an application of support vector machines in regression, is capable of handling nonlinear and high-dimensional data. The SVR model utilized a radial basis function kernel, with the following key hyperparameters:

- Penalty parameter *C* = 1.0: Controls model complexity to prevent overfitting.
- Epsilon *ε* = 0.2: Defines the width of the support vector boundary, influencing the model’s robustness.

#### 2.3.4 Random forest

RF is an ensemble learning method that constructs multiple decision trees and averages their outcomes to reduce overfitting. Key hyperparameters of the RF model included:

- Number of trees *n*_estimators_ = 30: Ensures sufficient model diversity.
- Maximum depth *max_depth* = 5: Limits tree depth to prevent overfitting.

#### 2.3.5 Extreme gradient boosting

XGB, a tree-based ensemble model, employs gradient boosting for efficient and accurate predictions. Its key hyperparameters were set as follows:

- Number of trees *n*_estimators_ = 30.
- Maximum depth *max_depth* = 4: Limits tree depth to prevent overfitting.
- Learning rate *learning_rate* = 0.1: Regulates each tree’s contribution; a smaller rate enhances generalization.
- Regularization parameters *reg_alpha* = 0.5 and *reg_lambda* = 0.5: Apply L1 and L2 regularization to prevent overfitting.

#### 2.3.6 Neural network

The NN model was constructed as a multilayer perceptron regressor with the following structure and parameters:

- Hidden layer size *hidden_layer_sizes* = (100),: One hidden layer with 100 neurons.
- Maximum iterations *max_iter* = 500: Restricts training time.
- Activation function: Rectified Linear Unit (ReLU) to enhance nonlinear representation.
- Learning rate: Adaptive learning rate to optimize training.

#### 2.3.7 Model training and validation

For LR and QCR, the models used average values of Chl, Flav, or NBI from the 1-5 LFT as single input variables. For PLS and machine learning models, four different variable combinations served as inputs:

- Combination 1: Chl measurements from the 1-5 LFT and their average (six variables).
- Combination 2: Flav measurements from the 1-5 LFT and their average.
- Combination 3: NBI measurements from the 1-5 LFT and their average.
- Combination 4: All variables from the three combinations above.

A 10-fold cross-validation was employed to randomly divide the dataset into training and validation sets, ensuring reliable and stable model evaluation. This method reduces the model’s dependence on data partitioning and enhances generalization.

### 2.4 Saturation effect analysis

To evaluate the saturation effects of different spectral measurements on rice nitrogen indices, we conducted an incremental change analysis that included the following steps:

#### 2.4.1 Data normalization

Normalization was performed using Min-Max scaling on both independent (*X*) and dependent (*Y*) variables to make them comparable.


$$X_{scaled}=\frac{X-X_{\min}}{X_{\max}-X_{\min}},\;Y_{scaled}=\frac{Y-Y_{\min}}{Y_{\max}-X_{\min}}$$

#### 2.4.2 Sorting

Data were sorted in ascending order based on *X*_scaled_ to facilitate the calculation of incremental changes, ensuring accurate reflection of *X’s* impact on *Y*.

#### 2.4.3 Increment calculation

We quantified the effect of *X*_scaled_ changes on *Y*_scaled_ by computing increments between adjacent data points.


$$\Delta X_{i}=X_{scaled,i+1}-X_{scaled,i},\;\Delta Y_{i}=Y_{scaled,i+1}-Y_{scaled,i}$$

#### 2.4.4 Threshold for Δ*X*

A minimum ∣Δ*X_i_* ∣ threshold of 1×10^−3^ was applied to exclude data points with very small Δ*X_i_* values. This step prevents excessively large rates of change in the subsequent calculations, ensuring stability and accuracy in the rate computations.


$$\Delta X_{i}=\{\begin{array}{c} \Delta X_{i},\;if\left|\Delta X_{i}\right|\geq 1\times 10^{-3}\\ NaN,\;if\left|\Delta X_{i}\right|<1\times 10^{-3} \end{array}$$

#### 2.4.5 Rate of change computation

The rate of change was determined to assess *Y_scaled_* variations relative to *X_scaled_*, with rates scaled to the range [-1, 1] using the maximum absolute rate.


$$Rate_{i}=\frac{\Delta Y_{i}}{\Delta X_{i}},\;Rate\_Scaled_{i}=\frac{Rate_{i}}{max\left(\left|Rate\right|\right)}$$

#### 2.4.6 Data smoothing

A moving average with a window size of 5 was used to smooth the rates, reducing random noise and emphasizing overall trends.


$${\mathrm{Rate\_Smoothed}}_{i}=\frac{1}{5}\sum_{j=i-2}^{i+2}{\mathrm{Rate\_Scaled}}_{j}$$

#### 2.4.7 Density curve analysis

To examine the behavior at high nitrogen levels, we focused on the top 20% of *Y_scaled_* values and used Gaussian Kernel Density Estimation (KDE) on the corresponding *X_scaled_* to obtain a smooth estimate of their probability density function. This non-parametric method does not assume any specific underlying distribution, making it suitable for real-world data. It helps in assessing whether the measurements continue to respond to increases in nitrogen levels or exhibit saturation.

### 2.5 Feature importance analysis and model evaluation

To gain deeper insights into the contributions of each input variable to the model’s predictions, we employed the SHAP (SHapley Additive exPlanations) method (Lundberg and Lee, 2017), which is based on game theory, to analyze feature importance for the RF and XGB models. SHAP values quantify the impact of each feature on the prediction outcomes by calculating its marginal contribution across all possible feature combinations. Compared to traditional feature importance metrics, the SHAP method offers consistency and additivity, enhancing model interpretability and providing guidance for optimizing measurement strategies in practical applications.

To comprehensively evaluate the predictive performance of each model, we used the coefficient of determination (R^2^), Root Mean Square Error (RMSE), and Relative Root Mean Square Error (RRMSE) as evaluation metrics. The formulas for these indicators are as follows:

• **Coefficient of determination**:


$$R^{2}=1-\frac{\sum_{i=1}^{n}{\left(Y_{i}-{\hat{Y}}_{i}\right)}^{2}}{\sum_{i=1}^{n}{\left(Y_{i}-\bar{Y}\right)}^{2}}$$

• **Root mean square error**:


$$RMSE=\sqrt{\frac{1}{n}\sum_{i=1}^{n}{\left(Y_{i}-\hat{Y}\right)}^{2}}$$

• **Relative root mean square error**:


$$RRMSE=\frac{RMSE}{\bar{Y}}\times 100\%$$

where *Y_i_* is the actual value, ${\hat{Y}}_{i}$ is the predicted value, $\bar{Y}$ is the mean of the actual values, and n is the number of samples.

### 2.6 Statistical analysis tools

All statistical analyses and model construction in this study were performed using Python 3.12.4. Data processing and analysis utilized libraries such as Pandas, NumPy, and SciPy. Machine learning modeling was done using Scikit-learn, XGB, TensorFlow, and Keras, while visualization was carried out using Matplotlib. Statistical tests were conducted with Statsmodels and SciPy.stats, and feature importance analysis was performed using the SHAP library. To ensure reproducibility of results, all random processes were set with the same random seed during modeling and testing.

## 3 Results

### 3.1 Relationship between Dualex measurements and rice nitrogen indicators

**Figure 1:**
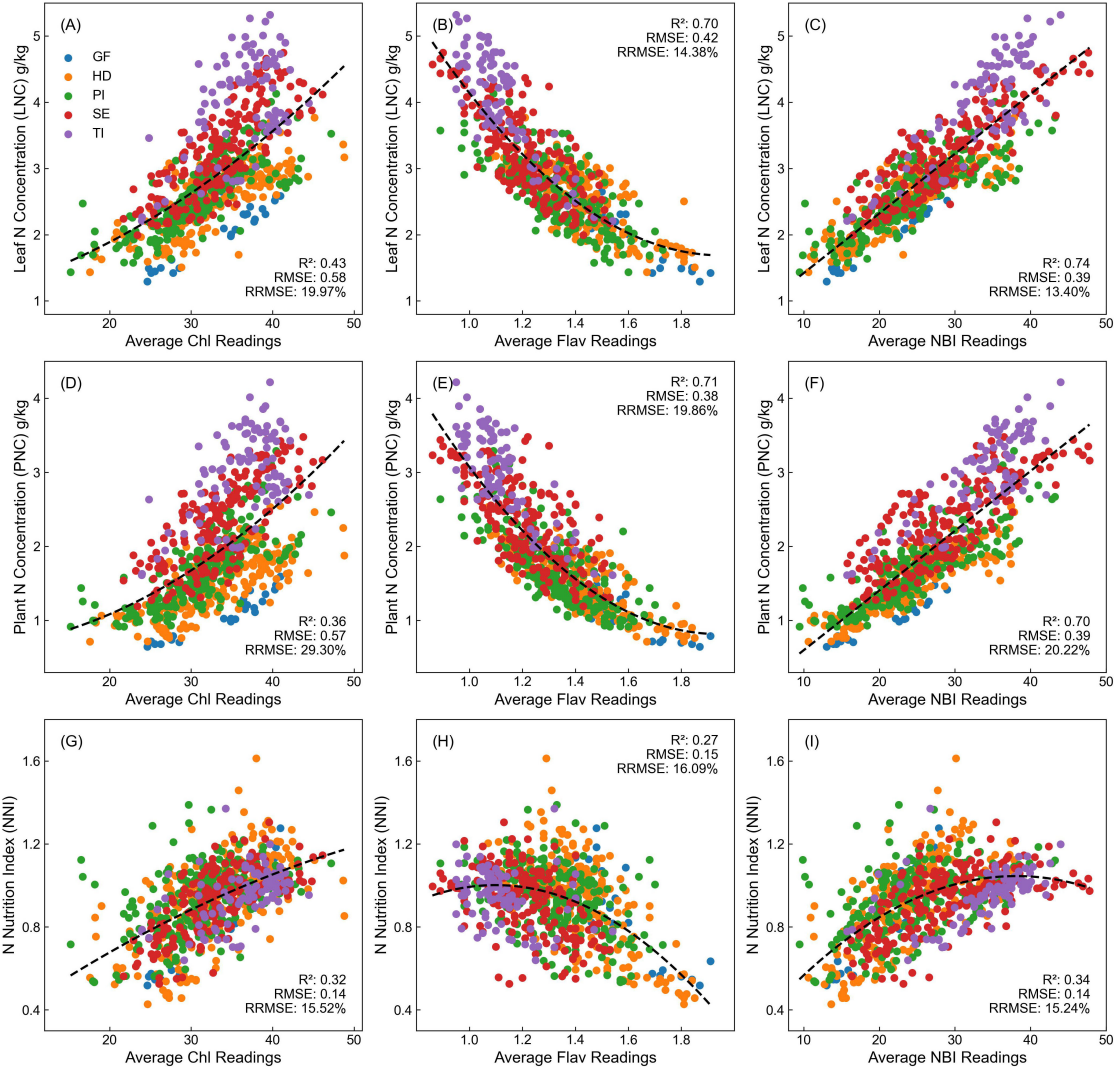
Relationship between Dualex values and rice nitrogen indicators across different varieties and growth stages. **(A–C)** show Chl, Flav, and NBI vs. LNC; **(D–F)** show Chl, Flav, and NBI vs. PNC; **(G–I)** show Chl, Flav, and NBI vs. NNI.

Average Dualex values (Chl, Flav, NBI) from the first to fifth LFT were analyzed using LR and QCR models to explore their relationship with rice nitrogen nutritional indicators—LNC, PNC, and the NNI (**Figure 1**). The findings revealed notable differences in the effectiveness of these indicators for nitrogen estimation depending on the specific Dualex metrics used.

Chl displayed a nonlinear relationship with both LNC and PNC (**Figures 1A, D**). As Chl levels increased, both LNC and PNC rose, showing accelerated growth rates at Chl values around 30. However, a saturation effect was evident between Chl values of 35 to 40, where further increases in Chl did not lead to corresponding changes in LNC and PNC.

Conversely, Flav showed a stable and linear relationship with LNC and PNC, without notable saturation effects (**Figures 1B, E**). Flav levels increased steadily as nitrogen content decreased, demonstrating a strong negative correlation with LNC and PNC across the full range, with high regression fits (R^2^ = 0.70 and 0.71). Even when accounting for differences across rice varieties and growth stages, Flav consistently provided an accurate assessment of nitrogen status.

NBI, defined as the ratio of Chl to Flav, exhibited a significant linear relationship with LNC and PNC (**Figures 1C, F**), achieving R^2^ values of 0.74 and 0.70, respectively. This underscores NBI’s robustness and stability in estimating nitrogen nutritional status. As a composite indicator, NBI effectively mitigates the saturation effect observed when using Chl alone, making it a highly reliable tool for nitrogen diagnosis.

For NNI estimation, which represents a relative value (the ratio of PNC to the N_c_), measurements across different growth stages showed consistent NNI values, primarily ranging between 0.4 and 1.4. However, Dualex readings varied across stages, leading to lower estimation accuracy for NNI (**Figures 1G–I**). Although NNI tended to increase with changes in Chl, Flav, and NBI, this trend exhibited significant variability across growth stages, resulting in inconsistent patterns.

### 3.2 Saturation effect analysis of Dualex values in estimating nitrogen indicators

**Figure 2:**
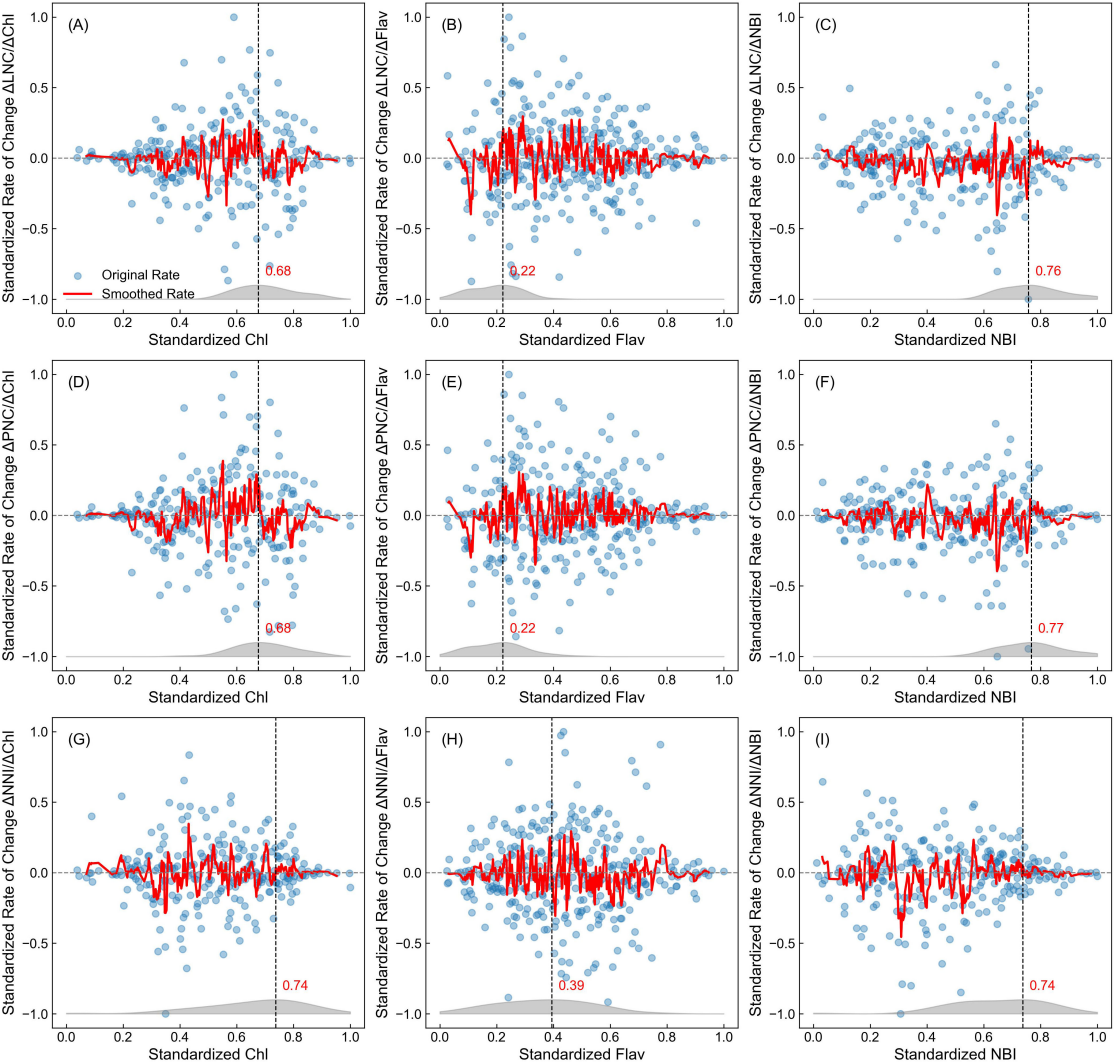
Standardized rate of change relative to Dualex values. This figure illustrates the standardized rate of change (ΔY/ΔX) of LNC **(A–C)**, PNC **(D–F)**, and NNI **(G–I)** in relation to standardized Dualex values: Chl, Flav, and NBI. In each subplot, blue scatter points represent the original calculated standardized rate of change data, while the red curve shows the smoothed trend line obtained using a 5-point moving average, reflecting the overall trend. The gray curve at the bottom indicates the density distribution of measurement values corresponding to the highest 20% of nitrogen indices (Y values). The vertical black dashed line marks the point of highest density, annotated with the corresponding X-axis value.

The saturation effect analysis of Dualex values in estimating rice nitrogen nutritional indicators is illustrated in **Figure 2**. To minimize the impact of model fitting differences when evaluating saturation effects across various Dualex indices, we applied an incremental analysis method using raw and standardized data. The standardized rate of change (ΔY/ΔX) was calculated based on these original data points. Each subplot displays the rate of change of nitrogen nutritional indicators relative to standardized Dualex values. In the analysis of Chl versus nitrogen indicators (**Figures 2A, D, G**), an initial low rate of change was observed in the lower Chl range, followed by a gradual increase, peaking at moderate Chl values. However, as Chl values continued to rise, the rate of change began to decline, particularly in the high Chl region. In the low Chl region, the low nitrogen indicator values were primarily responsible for the reduced rate, while in the high Chl region, a declining rate suggested that further increases in Chl no longer led to corresponding rises in nitrogen indicators, indicating a clear saturation effect. The density curves at the bottom further validated this: standardized Chl values peaked around 0.68 (for LNC and PNC) and 0.74 (for NNI), showing a marked decline in estimation accuracy at these levels.

In contrast, Flav exhibited a negative correlation with nitrogen indicators (**Figures 1B, E, H**). The rate of change remained relatively stable in the low Flav region (**Figures 2B, E, H**), indicating effectiveness in estimating nitrogen indicators. The density peaks for Flav values were observed at 0.22 (for LNC and PNC) and 0.39 (for NNI), with a gradual distribution, suggesting that even at high nitrogen indicator levels, Flav measurements were evenly spread without a significant saturation effect. However, for NNI, as Flav values decreased, a rapid decline in the rate of change was noted, with the density peak at 0.39 indicating a more pronounced saturation. Comparing this to **Figures 1H**, NNI exhibited almost horizontal distribution in the low Flav range, indicating diminished sensitivity to changes in Flav.

As a composite indicator, NBI (Chl to Flav ratio) displayed a more consistent trend in estimating LNC, PNC, and NNI (**Figures 2C, F, I**). The rate of change initially increased, then decreased with rising NBI, resembling the pattern seen in Chl but with density peaks closer to boundary values (0.76, 0.77, and 0.74, respectively). This suggests that NBI effectively estimates nitrogen nutritional status even at high nitrogen levels, exhibiting significantly less saturation than Chl alone.

In summary, Chl demonstrated a clear saturation effect, particularly at high nitrogen levels, where its sensitivity diminished. Conversely, Flav and NBI retained considerable variability in their rates of change at high nitrogen levels, showing enhanced sensitivity to changes in nitrogen status and providing a more accurate reflection of variations in rice nitrogen nutrition.

### 3.3 Model performance comparison for LNC, PNC, and NNI estimation

**Figure 3:**
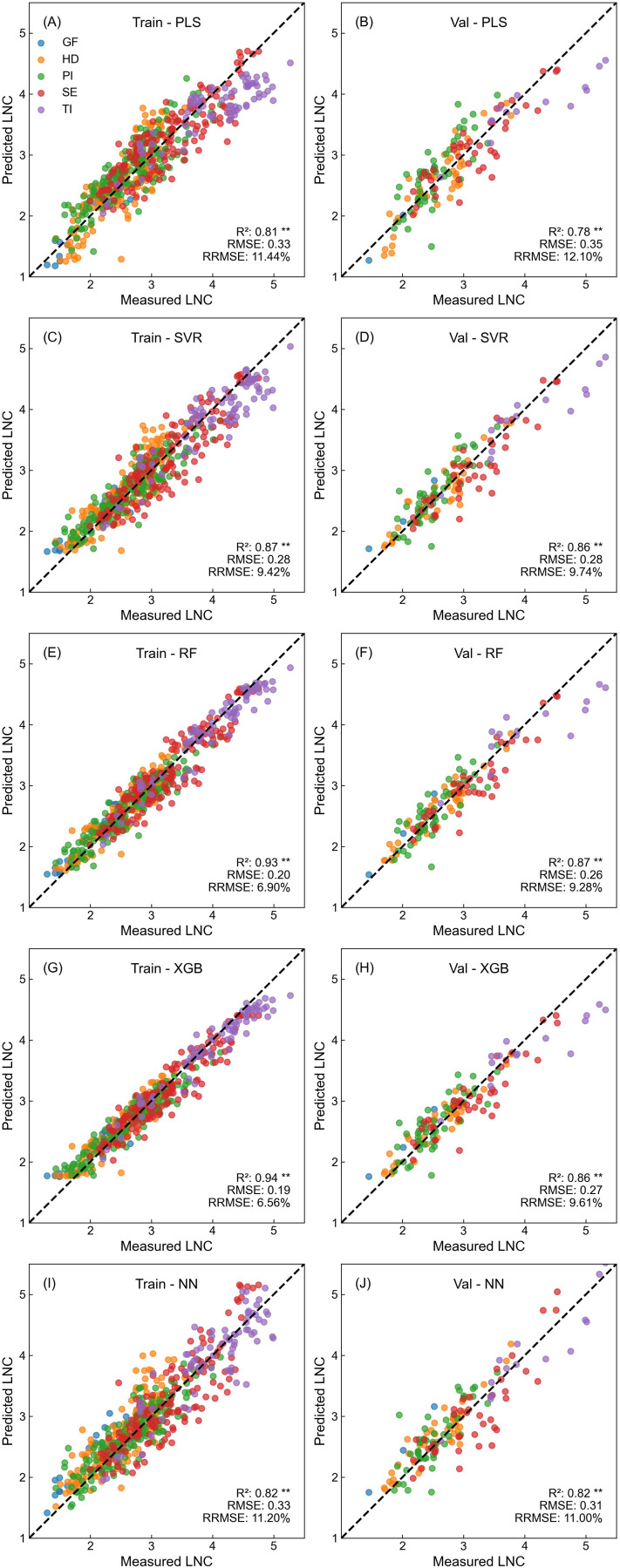
Performance of different machine learning models in predicting LNC based on Dualex readings. **(A, C, E, G, I)** show the training performance, and **(B, D, F, H, J)** show the validation performance for PLS, SVR, RF, XGB, and NN models, respectively.

**Figure 4:**
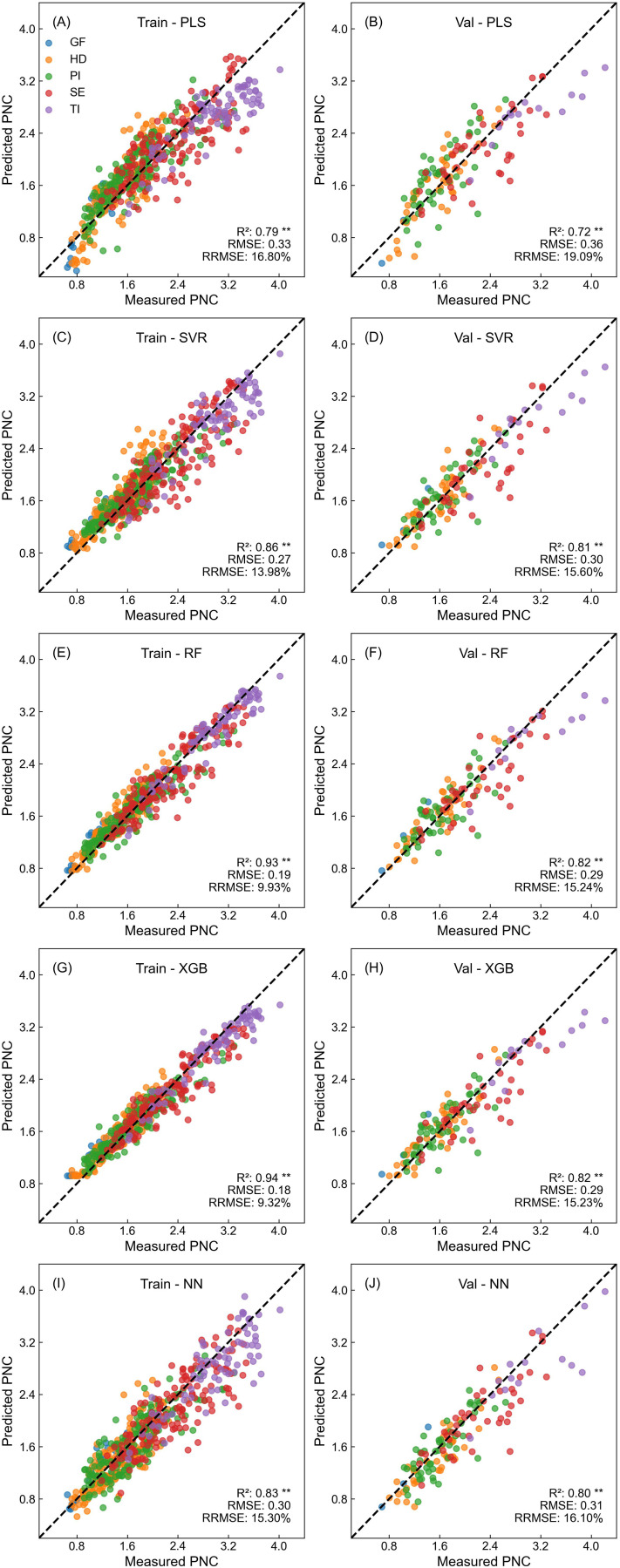
Performance of different machine learning models in predicting PNC based on Dualex readings. **(A, C, E, G, I)** show the training performance, and **(B, D, F, H, J)** show the validation performance for PLS, SVR, RF, XGB, and NN models, respectively.

**Figure 5:**
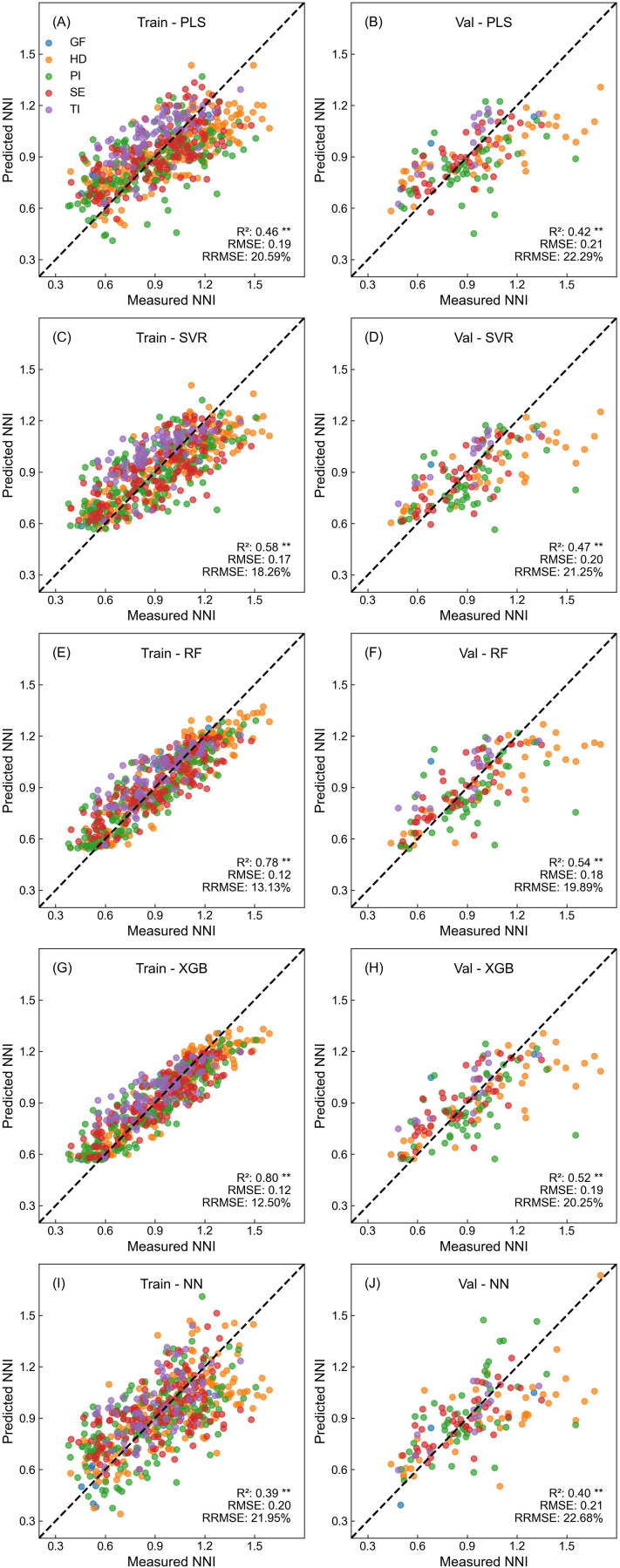
Performance of different machine learning models in predicting NNI based on Dualex readings. **(A, C, E, G, I)** show the training performance, and **(B, D, F, H, J)** show the validation performance for PLS, SVR, RF, XGB, and NN models, respectively.

**Figures 3**–**5** illustrate the performance of five different models in estimating rice nitrogen nutritional indicators using Dualex readings. Each model utilized measurements from the 1-5 LFT, including Chl, Flav, and NBI values for individual leaves and their averages, totaling 18 input variables. The models showed distinct differences in their performance across training and validation sets, particularly when estimating different nitrogen indicators. Overall, estimation accuracy was highest for LNC, followed by PNC, with NNI being the most challenging to estimate accurately.

For LNC estimation (**Figure 3**), all models demonstrated strong fitting accuracy, achieving R² values above 0.81 in training and 0.78–0.87 in validation datasets. SVR, RF, and XGB models demonstrated the highest validation accuracy, with R² values between 0.86 and 0.87, and RRMSE below 10%. Data from all sampling periods, except for minor overestimations at the tillering stage, were evenly distributed around the 1:1 line.

In estimating PNC (**Figure 4**), model performance was similar to that observed for LNC, despite with slightly reduced accuracy. RF and XGB remained the top performers, with validation R² values of 0.82 and RMSE at 0.29. The SVR model followed closely with an R² of 0.81. Conversely, PLS regression model demonstrated slightly lower performance.

For NNI estimation (**Figure 5**), all models showed a noticeable decline in fitting accuracy, which was consistent with regression analysis results from quadratic models. Despite the challenges, RF and XGB continued to lead, with validation R² values ranging from 0.52 to 0.54, marking a 71% improvement over QCR models. However, estimating NNI proved to be more difficult than LNC and PNC, with apparent biases in both low and high-value regions.

**Figure 6:**
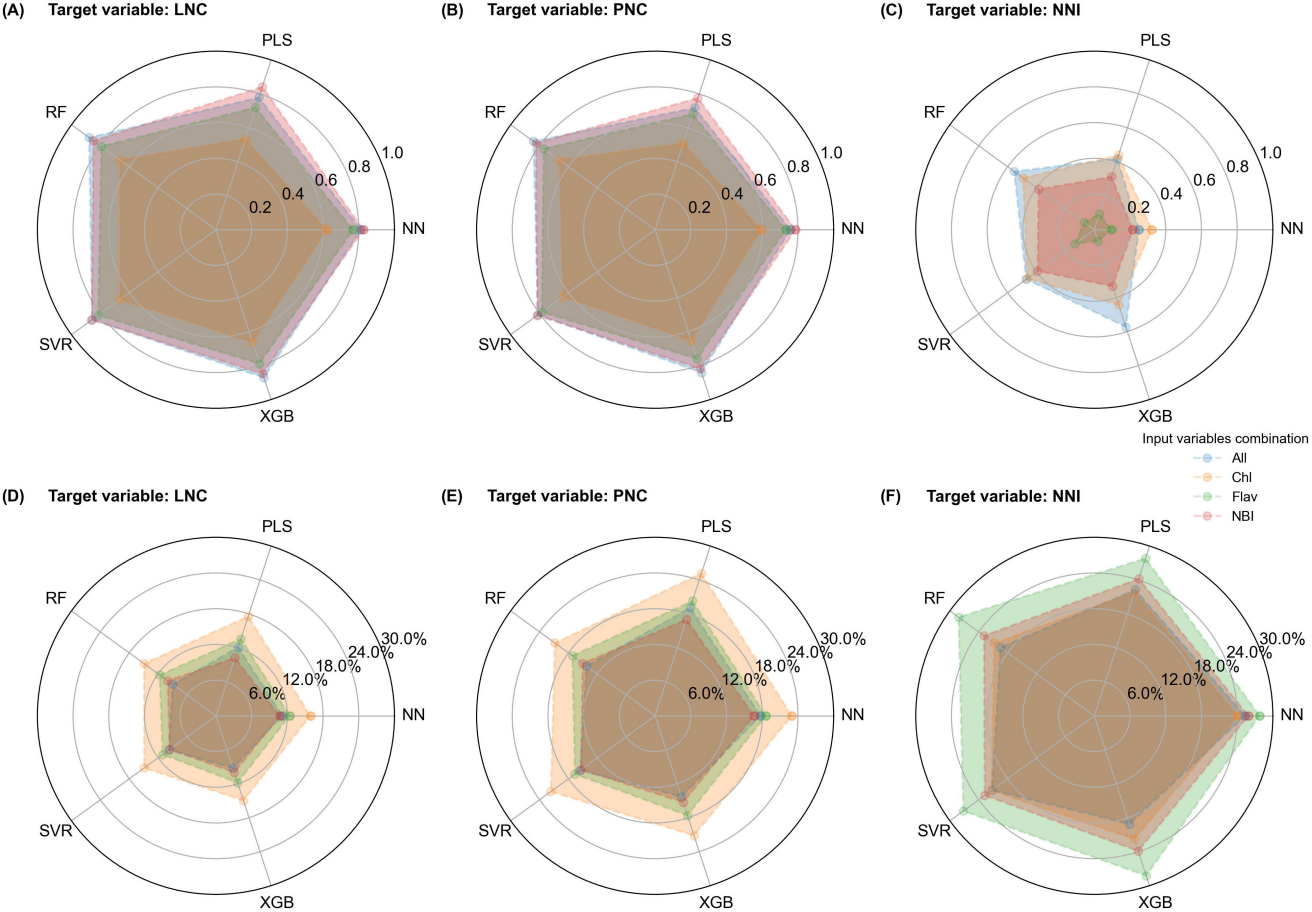
Comparison of machine learning model performance using different input variables for LNC, PNC, and NNI estimation. Each radar chart compares five models, with axes representing different input variables: All, Chl, Flav, and NBI. Charts **(A–C)** display R² values, and charts **(D–F)** show RRMSE for validation results.

Radar charts were used to visualize the estimation accuracy of different models and input variable combinations on the validation dataset (**Figure 6**). The R² values decreased progressively from LNC to PNC and then to NNI (**Figures 6A-C**). While LNC and PNC estimations showed near-equal accuracy, R² values for NNI dropped substantially. Across all input variable combinations, R² for NNI estimation declined by an average of 57% and 55% compared to LNC and PNC, respectively.

In comparing input variable combinations, the PLS model using multi-leaf NBI value was most effective in estimating LNC and PNC. For the other models, the all-variable combination (Combination 4) and NBI combination consistently provided high accuracy. Changes in RRMSE mirrored R² trends inversely (**Figures 6D-F**); models with higher R² exhibited lower RRMSE. RRMSE increased sequentially when estimating LNC, PNC, and NNI. Among the input variable combinations, models using only the Chl combination had the highest RRMSE for LNC and PNC estimations, indicating lower accuracy in these cases. In contrast, for NNI estimation, models using only the Flav combination had the highest RRMSE (**Figure 6F**). The NBI and all-variable combinations consistently achieved similar, superior accuracy across all nitrogen indicators.

### 3.4 SHAP analysis of feature importance in nitrogen estimation

**Figure 7:**
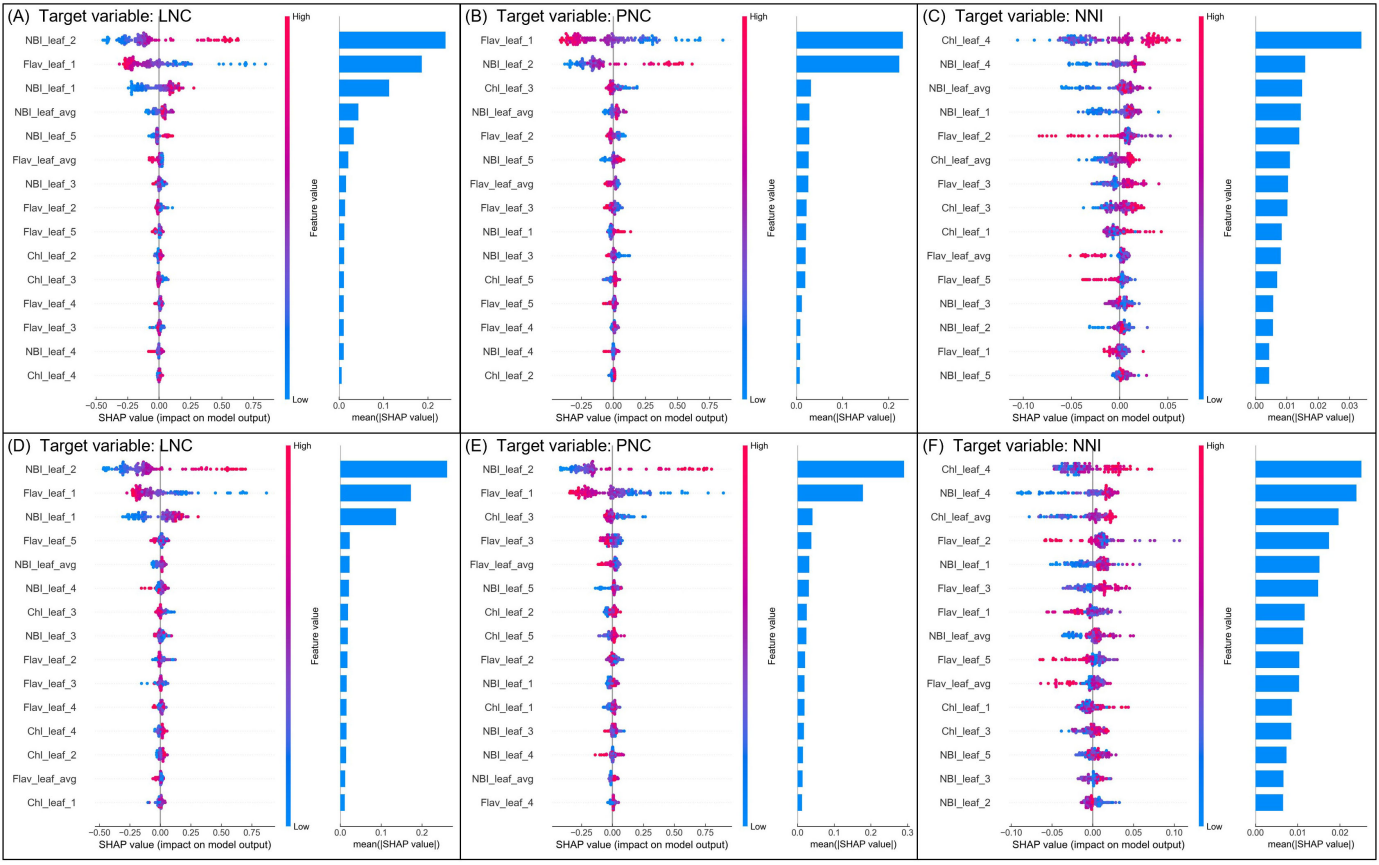
SHAP value analysis of feature importance for RF **(A–C)** and XGB **(D–F)** models in predicting rice nitrogen indicators. Each plot presents the SHAP values for the 15 most important features. In the SHAP scatter plots on the left, red indicates higher feature values and blue indicates lower feature values. Positive SHAP values indicate that the feature increases the predicted nitrogen indicator, while negative SHAP values indicate that the feature decreases it. The right side ranks feature importance based on the mean absolute SHAP value.

The feature importance analysis results based on SHAP values for the RF and XGB models in nitrogen estimation are presented in **Figure 7**. Both models used Combination 4 as input to assess the impact of each variable on their predictions.

For LNC prediction, **Figures 7A, D** show the SHAP analysis for the RF and XGB models, respectively. In the RF model (**Figure 7A**), the NBI value from the 2 LFT was the most influential variable, followed by the Flav value from the 1 LFT and the NBI value from the 1 LFT. The XGB model (**Figure 7D**) also highlighted these three variables as having the highest impact, with their SHAP values significantly surpassing those of other variables. This suggests that these variables were critical for accurate LNC prediction.

In the case of PNC estimation, **Figures 7B, E** illustrate the SHAP analysis for the RF and XGB models. The RF model (**Figure 7B**) indicated that the Flav value from the 1 LFT and the NBI value from the 2 LFT were the most significant variables. Similarly, in the XGB model (**Figure 7E**), the NBI of the 2 LFT and Flav of the 1 LFT were also identified as the most influential factors. This consistency across models underscores the importance of NBI and Flav as key indicators for both LNC and PNC estimation.

For NNI prediction, **Figures 7C**, F display the SHAP analysis results for the RF and XGB models. The RF model (**Figure 7C**) identified the Chl value from the 4 LFT as the most important variable, followed by the NBI value from the 4 LFT. The XGB model (**Figure 7F**) similarly showed these two variables as dominant; however, there were differences in their SHAP value distributions. In the RF model, the Chl value from the 4 LFT had a SHAP value notably higher than the other variables, whereas, in the XGB model, SHAP values of variables decreased in a stepwise manner, indicating that no single variable was overwhelmingly dominant.

Overall, the SHAP analysis results for LNC and PNC predictions revealed certain dominant variables that enabled the models to achieve high-precision estimates. However, for NNI prediction, the lack of clearly dominant variables and weaker associations between input variables and the target presented challenges in achieving satisfactory model accuracy.

## 4 Discussion

### 4.1 Saturation effect and its implications

Incremental analysis highlighted distinct differences in the saturation effects of different Dualex indices when estimating rice nitrogen indicators. The results showed that Chl experiences significant saturation at high measurement values, consistent with findings from studies using SPAD-502 (Yuan et al., 2016; Zhang et al., 2019a; Jiang et al., 2021) and chlorophyll-based multispectral/hyperspectral indices (Fu et al., 2021; Yao et al., 2023; Gao et al., 2024). Previous research has noted that SPAD values become less responsive to nitrogen at higher concentrations, complicating the differentiation of nitrogen levels (Yue et al., 2020). This saturation effect is documented across various crops, including rice (Wang et al., 2023), wheat (Jiang et al., 2021), and maize (Dong et al., 2021). Our findings reaffirm that Chl and SPAD measurements exhibit saturation under high nitrogen conditions.

When leaf chlorophyll content is low, an increase can enhance the leaf’s net photosynthetic rate (Tantray et al., 2020). However, as nitrogen availability rises and chlorophyll reaches a threshold level, photosynthetic rates plateau due to limits in light absorption area (Hou et al., 2019). At this point, excess nitrogen is allocated to non-photosynthetic reserves, altering the ratio of chlorophyll to leaf nitrogen (Hou et al., 2019; Mu and Chen, 2021). Consequently, while Chl is a sensitive indicator at low nitrogen levels, its effectiveness decreases at higher levels, introducing errors into predictive models. This trend was observed in our analysis, particularly in LNC predictions, where models relying solely on Chl values achieved lower R² scores, failing to capture nitrogen variations accurately. Therefore, Chl’s utility as a single diagnostic variable is limited, especially under high nitrogen conditions.

The saturation effect in this study was mainly observed when rice plants exhibited high nitrogen content. Since nitrogen concentration in rice leaves or plants gradually decreases during growth—a pattern known as the nitrogen dilution effect (Yao et al., 2021; Li et al., 2022a)—with the impact most noticeable around the tillering and jointing stages. This differs from canopy-level saturation observed after canopy closure, where high leaf area can cause remote sensing indices (e.g., NDVI) to saturate due to optical signal limitations, reducing their sensitivity to nitrogen changes (Tenreiro et al., 2021). Conversely, Chl saturation is primarily driven by physiological limits on chlorophyll content within leaves, necessitating a distinction between these two types of saturation in research.

In contrast, Flav and NBI showed no significant saturation effects, maintaining their effectiveness in estimating nitrogen indicators, even at high nitrogen levels. Flav is closely associated with plant stress responses, increasing under conditions of nitrogen stress (Li et al., 2021). When nitrogen is scarce, plants produce more flavonoids as a protective response (Gitelson et al., 2017). NBI combines the divergent responses of these indicators. In nitrogen-rich conditions, Chl levels are high while Flav remains low, leading to a high NBI. Under nitrogen deficiency, Chl decreases and Flav increases, resulting in a low NBI. By integrating complementary data from both indicators, NBI provides a robust reflection of nitrogen status, offering distinct advantages over Chl alone. This aligns with studies showing that NBI can overcome saturation and retain sensitivity under high nitrogen levels (Dong et al., 2022; Song et al., 2022). Our dataset, encompassing 15 rice varieties across different growth stages, confirmed that NBI and Flav maintained high predictive accuracy across various environmental conditions, suggesting these indicators can reliably reflect nitrogen status across diverse environmental conditions.

### 4.2 Comparison of machine learning models

We compared the performance of five machine learning models—PLS, SVR, RF, XGB, and NN—in predicting nitrogen indicators. Overall, XGB and RF models showed the highest accuracy for LNC, PNC, and NNI predictions, whereas PLS regression and SVR models had noticeably lower performance, lagging behind the tree-based approaches. This trend is consistent with findings from other studies comparing similar models (Fan et al., 2022; Kganyago et al., 2024). While PLS regression is effective at explaining linear relationships, it struggles with complex nonlinear patterns (Yang et al., 2023). SVR, though capable of managing nonlinear data, is highly sensitive to parameter selection and less efficient with high-dimensional inputs (Ben Ishak, 2016). Conversely, tree-based ensemble models like RF and XGB excel at handling nonlinear, high-dimensional datasets (Nzekwe et al., 2024), with XGB particularly excelling due to its gradient boosting optimization, which iteratively improves model performance.

The NN model, however, was less stable than the XGB and RF models in this study. This may be due to the relatively smaller dataset size, as neural networks generally require larger datasets to train effectively and achieve good generalization. While the NN performed adequately in estimating LNC and PNC, its predictions for NNI were less accurate than those of the other models. The RF and XGB models demonstrated strong performance, although the training R² values were slightly higher than the validation R² values, especially for NNI estimation, the differences were minimal. This highlights the importance of model selection and appropriate complexity in achieving reliable predictions (Moradi et al., 2020; Yates et al., 2023).

The lower estimation accuracy for NNI compared to LNC and PNC can be attributed to several factors. NNI is a composite index involving both PNC and N_c_, where N_c_ is dependent on plant biomass and derived from the nitrogen dilution curve. This adds layers of complexity and potential sources of error. The dynamic nature of biomass accumulation and nitrogen dilution across growth stages introduces variability that is difficult to capture with current models. Additionally, the relatively narrow range of NNI values reduces the models’ ability to distinguish subtle differences. Measurement errors in biomass and nitrogen content further compound the challenge. Future research could explore incorporating growth stage information, more precise biomass measurements, and additional physiological parameters to enhance NNI prediction accuracy.

### 4.3 Feature importance across different input variables and leaf positions

SHAP analysis was instrumental in interpreting the models by quantifying the contribution of each input variable to the predictions (Lundberg et al., 2017). This is essential for enhancing model transparency, which is particularly valuable in agriculture, where interpretable models facilitate practical application (Ryo, 2022). The SHAP results highlighted that spectral data from different leaf positions do not contribute equally to nitrogen prediction. For LNC and PNC, the most significant contributions came from measurements taken at the 1 LFT and 2 LFT, corroborating findings from related studies where spectral reflectance from top leaves was found to be most sensitive for nitrogen assessment (Dong et al., 2021; Lu et al., 2022; Wang et al., 2023). For NNI prediction, SHAP analysis pointed to the importance of Chl measurements from the 4 LFT, suggesting that chlorophyll content in middle to lower leaves is particularly relevant. This may stem from the generally lower estimation accuracy of NNI, which tends to make the significance of variables more uniform. Additionally, nitrogen translocation in middle and lower leaves closely aligns with the plant’s overall nitrogen nutritional status (Sun et al., 2018; Li et al., 2022b).

To improve NNI prediction accuracy, future strategies could include incorporating additional variables closely related to NNI, such as plant leaf area index (Tuccio et al., 2022), growth stages, and other physiological and environmental factors (Dong et al., 2022, 2024). Moreover, combining these models with crop growth simulations or radiative transfer models, as well as using multi-source data, could enhance the model’s ability to account for complex interactions (Raya-Sereno et al., 2024; Wang et al., 2024). Expanding the dataset’s size and diversity will also be crucial for improving generalization and predictive accuracy.

The differences in contributions from various leaf positions suggest the potential for optimizing measurement strategies in practical applications. By focusing on key leaf positions that provide the most significant input to the models, measurement efficiency can be improved. Reducing the number of measurement variables not only lowers costs but also enhances the computational efficiency of the models, making them more viable for real-world use.

## 5 Conclusion

This study systematically assessed the effectiveness of Dualex measurements for estimating rice nitrogen nutritional indicators. The results indicated that Chl experiences significant saturation at high nitrogen levels, limiting its utility as a nitrogen diagnostic indicator. In contrast, Flav and NBI did not exhibit saturation effects and remained highly sensitive under high nitrogen conditions, accurately reflecting rice nitrogen status. Among the machine learning models tested, RF and XGB were the most effective in predicting LNC and PNC. SHAP analysis identified the most influential feature variables and leaf positions, further enhancing model interpretability and accuracy.

Future research should focus on expanding the dataset to include more diverse rice varieties and growth stages and incorporating additional crop and environmental variables. Additionally, exploring the applicability of Flav and NBI across different crops and environmental contexts could provide broader technical support for nitrogen management in precision agriculture.

## Data Availability

The original contributions presented in the study are included in the article/supplementary material. Further inquiries can be directed to the corresponding authors.
